# O038: Automated/electronic systems for hand hygiene monitoring: a systematic review

**DOI:** 10.1186/2047-2994-2-S1-O38

**Published:** 2013-06-20

**Authors:** L Arnoldo, D Pittet, J Boyce, H Sax, B Allegranzi

**Affiliations:** 1 University of Udine, Udine, Italy; 2 University of Geneva Hospitals, Geneva, Switzerland; 3 Yale-New Haven Hospital, New Haven, USA; 4 Zurich University Hospitals, Zurich, Switzerland; 5 World Health Organization (WHO), Geneva, Switzerland

## Introduction

Automated/electronic monitoring systems (AEMS) of hand hygiene (HH) indicators are now available.

## Objectives

We evaluated technologies used and evidence regarding their validity, suitability for use and advantages compared to gold standard methods.

## Methods

We conducted a systematic review of the literature searching the Cochrane Library, PubMed and EMBASE up to Feb. 2013, with no language or time restriction. All studies (observational & interventional) using AEMS were selected.

## Results

The search yielded 341 abstracts. Of 29 selected articles, 19 were included in the review. Of these, 17 studies were conducted in high-income countries, mostly in teaching hospitals (11). Technologies used were: automated count dispensers (7); automated count dispensers associated with either system detecting entries/exit (5) or electronic personal badge (2), or system activated by the nurse (1); electronic personal badge for alcohol vapor detection (2) or entries/exits detection (1); video systems (2). In studies evaluating HH compliance (9), standard definitions of opportunities for HH (OHH) were used in 1 study only. Types of OHH were: room entry and/or exit (10) and WHO Moments 1 and 4 (1). Among studies comparing HH compliance measured by AEMS with direct observation (6), 2 evaluated the concordance between methods (95% and 64%).

## Conclusion

Strengths of AEMS are the possibility of continuous monitoring and automatic data download and analysis, mitigation of the Hawthorn effect and minimal requirement of human resources. Limitations of AEMS tested were lack of standard definitions of OHH, and inability to identify healthcare workers and to evaluate HH technique and glove use. Most AEMS did not measure HH compliance and limited evidence is available to validate their use compared to direct observation. Finally, their cost-effectiveness remains unknown and suitability for use in settings with limited resources is unlikely. These new technologies are promising, provided that they reflect the WHO 5 moments for HH, but additional research is needed to support their adoption as a standard.

## Disclosure of interest

None declared.

